# Coronary flow and reactivity, but not arrhythmia vulnerability, are affected by cardioplegia during cardiopulmonary bypass in piglets

**DOI:** 10.1186/1749-8090-8-157

**Published:** 2013-06-19

**Authors:** Petru Liuba, Sune Johansson, Erkki Pesonen, Michal Odermarsky, Axel Kornerup-Hansen, Anders Forslid, Elhadi H Aburawi, Thomas Higgins, Malene Birck, Valeria Perez-de-Sa

**Affiliations:** 1 Division of Cardiology, Children’s Heart Center, Skåne University Hospital, Lund, Sweden; 2 Division of Cardiac Surgery, Children’s Heart Center, Skåne University Hospital, Lund, Sweden; 3 Division of Anesthesiology and Intensive Care, Children’s Heart Center, Skåne University Hospital, Lund, Sweden; 4 Department of Animal Science, Lund University, 22185 Lund, Sweden; 5 Department of Veterinary Disease Biology, Faculty of Life Sciences, University of Copenhagen, Copenhagen, Denmark; 6 Department of Paediatrics, College of Medicine and Health Sciences, UAE University, Al Ain, UAE

**Keywords:** Cardiopulmonary bypass, Coronary, Cardioplegia, Piglets

## Abstract

**Background:**

Surgery under cardiopulmonary bypass (CPB) is still associated with significant cardiovascular morbidity in both pediatric and adult patients but the mechanisms are not fully understood. Abnormalities in coronary flow and function have been suggested to play an important role. Prior studies suggest protective effects on coronary and myocardial function by short intravenous (i.v.) infusion of cyclosporine A before CPB.

**Methods:**

Barrier-bred piglets (10–12 kg, n=20) underwent CPB for 45 min, with or without antegrade administration of cardioplegic solution. Prior to CPB, half of the animals in each group received an i.v. infusion of 100 mg/kg cyclosporine A. The left anterior descending coronary flow velocity responses to adenosine, serotonin, and atrial pacing, as well as left ventricular function and postsurgical vulnerability to atrial fibrillation (Afib) were assessed by intracoronary Doppler, epicardial echocardiography, and in vivo electrophysiological study, before and 8 hours after surgery. Plasma C-reactive protein (CRP) and fibrinogen were measured at both time-points.

**Results:**

Cyclosporine infusion did not influence any of the studied variables (p>0.4). Coronary peak flow velocity (cPFV) rose significantly after surgery especially in the cardioplegia group (p<0.01 *vs*. non-cardioplegia group and pre-surgery). cPFV responses to adenosine, but not to serotonin, tended to decrease (p=0.06) after surgery only in cardioplegia group (p=0.06; p=0.8 in non-cardioplegia group vs pre-surgery). Also, cPFV response to atrial pacing was lower in the cardioplegia than in the non-cardioplegia group (p=0.02). Neither vulnerability nor duration of induced Afib after CPB differed between groups (Chi-square p=0.4). Cyclosporine had no significant effect on coronary indexes or arrhythmia vulnerability (p>0.4). There was no difference in systolic myocardial function between groups at any time point.

**Conclusion:**

In piglets, CPB with cardioplegia was associated with profound abnormalities in coronary vasomotor tone and receptor-related flow regulation, whereas arrhythmia vulnerability appeared to be comparable with that in non-cardioplegia group. In this study, preconditioning with cyclosporine had no detectable protective effect on coronary circulation or arrhythmia vulnerability after CPB.

## Background

The survival and quality of life of patients with congenital heart disease (CHD) undergoing surgery with cardiopulmonary bypass (CPB), even with very complex cardiac malformations, has improved significantly during the past decade. Nevertheless, surgery with CPB remains associated with appreciable cardiovascular morbidity in both pediatric and adult patients [1]. Recent clinical studies have highlighted several modifiable and nonmodifiable risk factors which could affect the cardiac function; intraoperative variables such as duration of CPB and aortic cross-clamping, type and temperature of cardioplegic solution seem to significantly influence the postoperative outcome [1,2]. Although the mechanisms whereby these different factors, together or independently, influence cardiovascular function remain unclear, perioperative changes in coronary flow and function might be a common pathway [3].

A prior study suggested that coronary vasomotor function is altered by cold crystalloid cardioplegia [4], but to a less extent by warm blood cardioplegia [5]. These changes appear to involve both the microvascular [6] and epicardial [7] coronary bed with significant coronary endothelial damage and apoptosis. A study by Oka *et al*. [8] suggested that addition of cyclosporine A to the cardioplegic solution could alleviate cardiomyocyte’s mytochondrial dysfunction and hence protect the myocardium against CPB-related ischemic injury.

We have earlier demonstrated that coronary flow is altered after surgery with CPB in children with CHD [9]. The observation of increased coronary blood flow after surgery with CPB, particularly in the setting of impaired coronary vasomotor function, should intuitively suggest decreased coronary flow reserve, which has been speculated to contribute to myocardial dysfunction and arrhythmias postoperatively [10]. To our knowledge, the relationship between coronary vasomotor function and arrhythmia vulnerability in the early postoperative period has not yet been investigated.

In the present study, we investigated whether administration of normothermic blood cardioplegia influence coronary and myocardial function after CPB in young miniaturized pigs, and whether these changes could be prevented by preoperative intravenous infusion of cyclosporine A. The threshold of postoperative tachyarrhythmia was also studied.

## Methods

Twenty barrier-bred, specific-pathogen-free, 8-9-week old, Göttingen minipigs (Ellegaard Göttingen Minipigs, Dalmose, Denmark), housed in a barrier-protected animal facility under controlled conditions (temperature between 18 and 22°C and relative humidity 30–60%) were allowed to acclimatize for 1 week before the start of the study. All animals received proper care in compliance with the Swedish Ethics Guidelines for the use of laboratory animals.

On the study day, the animals were randomized to one of the study groups and sedated with a mixture of ketamin and midazolam (10 mg/kg and 1 mg/kg, respectively) given intramuscularly before a 22 G cannula was inserted into an ear vein. Under sedation with propofol i.v. (approximately 0.5 - 2 mg/kg), the animals were orally intubated with a cuffed endotracheal tube (size 3.5 - 4.5). Mechanical ventilation was then started with oxygen-enriched room air (air and pure O_2_ in ratio of 2.2:1; 8 mL/kg, 30 breaths/min, 5 PEEP) using a Siemens SERVO 300 respirator (Siemens, Erlangen, Germany).

The animals were subsequently placed prone on an X-ray table and monitored with pulse oximetry (% Sp_O2_) and a 3-lead EKG. Central temperature was monitored by a rectal probe. Through surgical cutdown, a 5F double-lumen central venous catheter (COOK Medical Inc, IN, USA) was inserted into the left internal jugular vein for drug administration and central venous pressure monitoring, and a 20 G cannula was inserted into the left carotid artery for blood pressure monitoring and blood sampling. A baseline blood sample was taken and preserved in ice for C-reactive protein, plasma lipids and fibrinogen analysis. Intracoronary doppler velocimetry was thereafter performed according to the protocol described below.

Following coronary assessment, the animals received for 10 minutes an intravenous infusion of either cyclosporine A 100 mg/kg or saline via the central venous line. Cardiac rhythm monitoring with a portable Holter system was performed for 5 minutes preoperatively and continued immediately after surgery until the end of the postoperative coronary assessment (8 hours after surgery).

### Coronary velocimetry protocol

The coronary flow velocity study was performed before and 6–8 hours after completion of surgery [11,12]. After an intravenous bolus of 100 IU/kg heparin, a 4F short sheath was inserted into the right carotid artery and a 4F Judkins right coronary catheter was advanced into the ostium of the left coronary artery (LCA) under fluoroscopy guidance. A Volcano ComboWire (Pressure/Flow Guide .014″ Wire, Volcano, CA, USA) connected to a real-time spectrum analyzer (ComboMap System, model 6800, Volcano, CA, USA), was then inserted through the catheter and positioned in the mid-part of the left anterior descending coronary artery (LAD). Coronary averaged peak velocity (APV) and pressure were displayed continuously on the system’s monitor. Data were stored on the hard disk for off-line analysis. After stabilization of the baseline Doppler signal, intracoronary adenosine (5 μg/kg), bradykinin (10^−6^ M), and serotonin (10^−6^ M) were each given as a bolus through the catheter and the peak averaged velocity assessed. All drugs were given in succession 10 min apart, and in the same order, in all animals. Complete recovery of coronary flow velocity and pressure (ie, steady state) was attained in each animal before the next drug was given.

Before administration, all drugs were diluted with 0.9% saline to a volume of 3 ml. A recovery period of 10 min was allowed between drug administration until the coronary flow velocity values returned to baseline.

Postoperatively, coronary velocity response to incremental increases in heart rate (20 beats/minute every 2 minutes up to 60 beats/minute over the baseline heart rate) was assessed as well.

### Echocardiography

Epicardial echocardiography was performed before the start of CPB (after thoracotomy) and 6–8 hours after CPB, immediately before the postoperative coronary flow study. A Philips ultrasound system (Royal Philips Electronics, Amsterdam, The Netherlands) equipped with a 7-MHz probe was used. Standard 4- and 5-chamber apical and short-axis parasternal views were obtained in all animals. Left ventricle’s (LV) and aortic valve dimensions, aortic outflow signal and mitral inflow signal were assessed by Doppler as earlier described [13]. All images were stored on the system’s hard disk for later analysis of LV area and volume, systolic function (ejection fraction and shortening fraction), and diastolic function (mitral E/A ratio, total and “E”/“A”VTI).

### CPB

Through a median sternotomy, the pericardium was opened, and the aorta and caval veins cannulated after heparinization (300 U/kg). CPB, using a centrifugal pump (Bio-Medicus, Medtronic USA inc., MN, USA) and a pediatric membrane oxygenator (Minimax Plus, Medtronic USA inc., MN, USA), was instituted with a flow rate of 2.5-3 L/min/m2, and the animals were systemically cooled to 32 degrees Celsius. The system was primed with 500 mL of whole blood stabilized with citrate (Veterinary Pharmacy, University of Copenhagen, Denmark) sampled 3–6 days prior to surgery from slaughter pigs anaesthetized with isoflurane (Baxter, Allerød, Denmark). The hematocrit was maintained between 25% to 30%, and the mean arterial pressure around 60 mm Hg. CPB was carried out for 45 min, either without (n=10) or with antegrade administration for 20 minutes of blood cardioplegia (n=10). The ascending aorta was thereafter cross-clamped, and a double-lumen aortic root cannula inserted for antegrade delivery of cardioplegic solution. Cardioplegia was given via a roller pump that mixed oxygenated blood with modified St Thomas’ cardioplegic solution in a 4:1 ratio (30 ml/kg), at a pressure of ca 60 mm Hg. The cardioplegic solution was kept at room temperature before delivery. During weaning from CPB, all animals were started on dopamine 5 μg/kg/min, milrinone 0.5 μg/kg/min (maintenance dose), and 5% albumin (50 mg/ml, CSL Behring, Danderyd, Sweden) 25 ml/hour in order to maintain stable hemodynamics. Epicardial electrodes were placed in standard position (2 on the right atrium and 2 on the right ventricle) for later cardiac pacing during coronary velocity assessment.

#### Statistics

All coronary responses to vasoactive agonists and cardiac pacing are expressed as a ratio of peak (after drug infusion) to baseline (before drug infusion) velocities (peak-to-baseline). Significant differences between groups in numerical variables were calculated with ANOVA followed, when indicated, by Bonferroni post-hoc test. To reduce the impact of scattering and skewness, given the small number of animals, all numerical variables were log-transformed. Differences were considered statistically significant at *P* < 0.05. Statistical analysis was carried out with SAS 9.1 (SAS Institute, Cary, NC). Data are presented as means and SD. Mean arterial pressure and blood gases (O_2_ and CO_2_) were maintained within normal range in all animals postoperatively.

## Results

Two animals died postoperatively, one from the noncardioplegia&cyclosporine group due to ventricular fibrillation (no resuscitation attempted) and the other one from the cardioplegia & non-cyclosporine group due to recurrent massive bleeding. All data from these animals were therefore discarded.

### Cyclosporine on post-CPB variables

Cyclosporine had no significant effect on coronary indexes or arrhythmia vulnerability (p>0.4). Neither inflammatory or lipid variables were influenced by cyclosporine (p>0.5).

There were no significant differences in MAP or blood gases between treatment groups (p>0.4). All animals were in sinus rhythm after CPB. Holter analysis during the postoperative period revealed short-lasting, hemodynamycally insignificant episodes of atrial-ventricular block II, supraventricular tachycardia and atrial fibrillation/flutter (AFib/AF) in nearly all animals. The frequency and duration of AFib/AF were similar in all groups (p>0.2).

### Inflammatory and lipid data

Neither C-reactive protein (CRP) nor fibrinogen concentrations differed between the groups at any timepoint (p>0.05). Among lipid variables, HDL dropped significantly after CPB in the cardioplegia group (p=0.03) but remained relatively unchanged in the noncardiopegia group (p>0.1). Cyclosporine had no influence on any of these variables (p>0.2).

### Coronary peak velocity assessment

**Figure 1:**
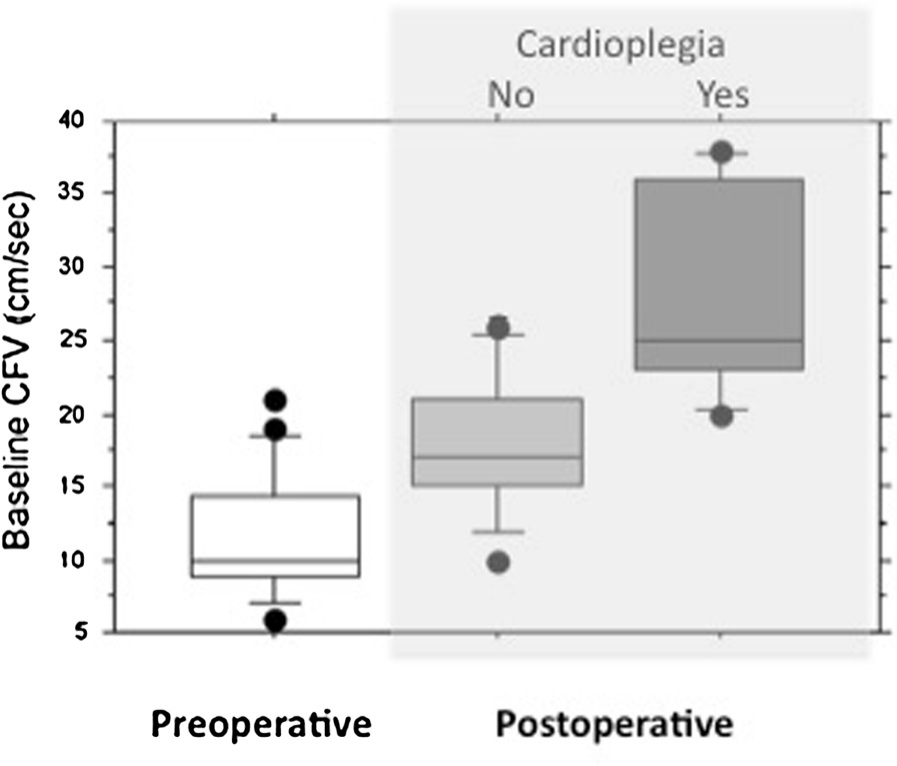
Baseline averaged coronary flow velocities in animals before (empty bar), and after surgery with (light gray bar) and without cardioplegia (dark gray bar).

**Figure 2:**
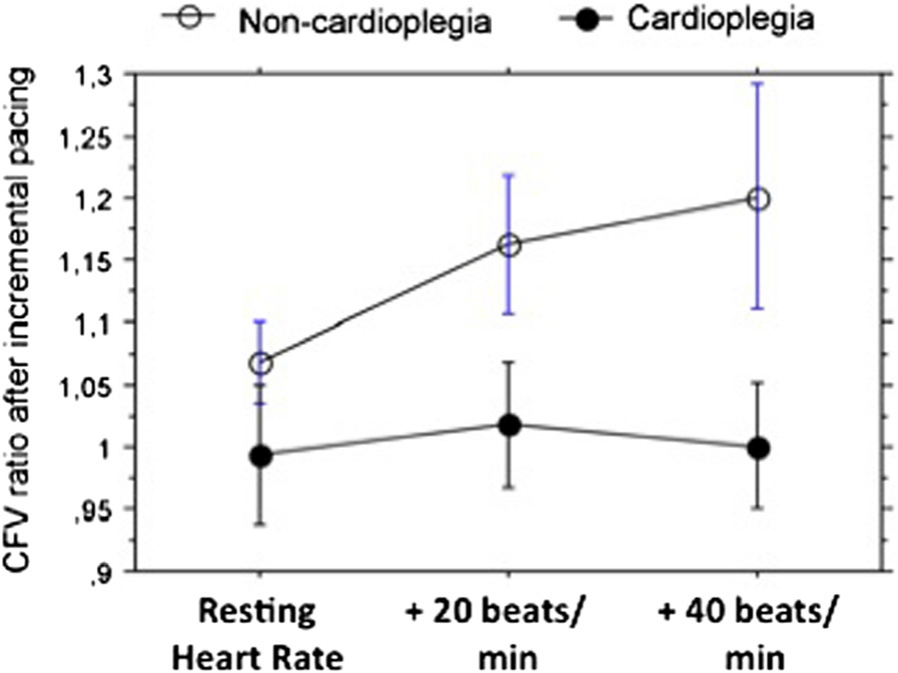
Peak coronary flow velocity response (expressed as peak-to-baseline ratio) to incremental increase in heart rate via cardiac pacing after surgery with (empty squares) and without (solid squares) cardioplegia.

Coronary peak flow velocity (cPFV) rose significantly after surgery in both cardioplegia and noncardioplegia animals (p<0.01 vs. pre-surgery for both groups), with the largest velocity increase in the cardioplegia group (p=0.001 vs noncardioplegia group; Figure 1). cPFV responses to adenosine, but not to serotonin (p>0.2), tended to decrease after surgery only in the cardioplegia group (p=0.06). In contrast, cPFV response to atrial pacing was lower in cardioplegia than in non-cardioplegia animals (p=0.02; Figure 2).

### Arrhythmia threshold

Afib was induced in all animals postoperatively at a threshold of 180–200 msec. However, neither this nor the duration of induced Afib differed between groups (p=0.4).

### Epicardial echocardiography data

LV end-diastolic diameters were significantly larger after CPB (p=0.03), whereas postoperative EF and SF were comparable to preoperative values (p>0.2). Similarly, CO and mitral inflow variables (E/A, total velocity time integral (VTI), and VTI“E”/VTI“A”) remained unchanged as compared to preoperative values (p>0.1).

## Discussion

**Figure 3:**
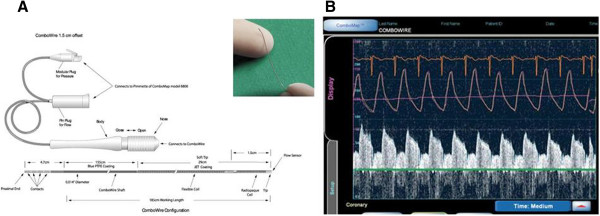
**Illustration of Volcano ComboMap system and Combowire.** Panel **A**: Volcano ComboMap® screenshot showing both pressure and flow measurements in coronary circulation; Panel **B**: illustration of Combowire used for intracoronary flow and pressure assessment.

Using reproducible and sensitive in-vivo methods for evaluation of coronary vasomotor and cardiac function [13,14], we aimed to assess whether the use of tepid blood cardioplegia during CPB of miniature piglets would affect the heart and the coronaries during the first hours after surgery, and whether preconditioning with cyclosporine A could alleviate the putative negative impact of cardioplegia on these indexes. Inflammatory and lipid indexes were also assessed. Using several vasoactive agonists known to dilate the coronaries through separate molecular endothelium-dependent and independent mechanisms, we observed cardioplegia-related abnormalities in coronary vasomotor tone and reactivity 6 to 8 hours post-CPB, though both the inflammatory markers (C-reactive protein and fibrinogen) and the myocardial function (assessed by epicardial standard echocardiography) were comparable to preoperative values. Furthermore, blood cardioplegia did not seem to influence in any way the susceptibility to postoperative arrhythmias, as demonstrated by the equal frequency of spontaneous and stimulator-induced rhythm disturbances in the cardioplegia and non-cardioplegia groups. No significant preconditioning effect of cyclosporine on coronary function was demonstrated. In the present study, the coronary flow measurements were achieved using the Volcano Combomap® system, which has the advantage of assessing simultaneously both pressure and flow velocity (Figure 3/Panel A) via a 0.014″ coronary wire (Figure 3/Panel B). Our group has a certain expertise with using this device in the swine model [11,12].

Cardiopulmonary bypass (CPB) remains a central part of surgery of congenital heart defects in both pediatric and adult patients [1]. In spite of major technical improvements in the performance of CPB during the past two decades, cardiovascular complications, particularly during the early postoperative period, are still present. While postoperative tachyarhythmias, often in form of atrial fibrillation (AFib), are more common in adults, heart failure and vascular complications, such as pulmonary and systemic hypertension, appear to be less age-related, with a greater prevalence among patients with depressed cardiac function at the time of surgery [1,2,15]. Prior in-vitro studies have provided suggestive evidence that some of these complications might relate to profound coronary and systemic vascular dysfunction that occurs during reperfusion [4-7]; others [8,16] have suggested potential benefit in partially reversing these effects by substances with protecting effects of vascular endothelium and cardiomyocites (pre- or postconditioning).

The increase in coronary flow after cardiac surgery has been demonstrated both in animal and clinical studies [9,17], but the replication of this phenomenon in the present study is a key finding, since tepid blood was used instead of cold crystalloid cardioplegia. The mechanisms might relate to elevated circulating levels of cytokines (not measured in this study), in particular tumor necrosis factor-alpha (TNF- α), and complement activation [18], which are triggered mainly through the contact between the blood and the foreign surface of the CPB circuit. TNF-α upregulates inducible nitric oxide (NO) synthase activity with secondary increased release of NO, thereby contributing to the reduction in the intrinsic tone of the coronary microcirculation [19]. Increased amounts of intravascular NO lead to formation of peroxynitrate, which is highly damaging for endothelial cells [20]. This effect is further exacerbated by ischemia-reperfusion injury [21]. The involvement of the coronary endothelium is also suggested by the difference in coronary velocity response to cardiac pacing, which is known to cause dilatation through the release of endothelial NO [22,23]. Dysfunction of coronary smooth muscle cells might occur as well, as indicated by the mildly impaired coronary response to adenosine.

In the present study, preconditioning with cyclosporine A prior to CPB conferred no observable protection on either coronary flow or function postoperatively. Although, in high doses, ie, following cardiac transplantation as an immunosuppressive agent, cyclosporine is known to deter coronary endothelial function [24], when used in lower doses for short periods of time or as addition to the cardioplegia solution, it appears to exert favourable effects on both the cardiomyocytes and coronary vascular function [8,25]. Cyclosporine A has been reported to ameliorate reperfusion injury in several in vitro and in vivo animal models of unprotected and protected ischemia [26,27]. In a neonatal piglet model of cardioplegic arrest [8], cyclosporine A was shown to prevent apoptosis-related mytocondrial dysfunction. Similar effect of cyclosporine A on mitochondria was demonstrated in a rabbit model of ischemia-reperfusion, and was associated with improved post-cardioplegia myocardial performance [28]. Therefore we decided to test its eventual efficacy in protecting the coronary function in this animal model. The lack of effect in the present study could be explained in part by the relatively mild coronary dysfunction, which, perhaps in conjunction with the postoperative use of milrinone [29], another drug with important vasoprotective action, might have concealed a potential effect of cyclosporine. The preserved postoperative myocardial function, documented by echocardiography, could support this assumption.

## Conclusions

In conclusion, cardioplegia with tepid blood during surgery with CPB in piglets was associated with abnormalities in coronary vasomotor tone and receptor-related flow regulation, whereas myocardial function appeared to be preserved. In this study, preoperative administration of cyclosporine had no observable protective effect on coronary circulation or arrhythmia vulnerability after CPB.

## Competing interests

The authors declare that they have no competing interests.

## Authors’ contributions

PL designed the study, performed the coronary Doppler study, contributed to the statistical analysis, and drafted the manuscript. SJ performed the cardiac surgery. EP contributed to the coronary Doppler study. MO participated in the analysis of the coronary and heart rhythm data. TH and EA performed the cardiac ultrasound study. VP, AKH, AF, and MB were responsible for sedation and anaesthesia, and the periperative care of animals. All authors read and approved the final manuscript.
